# Atomicity and Regularity Principles Do Not Ensure Full Resistance of ECC Designs against Single-Trace Attacks †

**DOI:** 10.3390/s22083083

**Published:** 2022-04-18

**Authors:** Ievgen Kabin, Zoya Dyka, Peter Langendoerfer

**Affiliations:** 1 IHP—Leibniz-Institut für Innovative Mikroelektronik, 15236 Frankfurt, Germany; dyka@ihp-microelectronics.com (Z.D.); langendoerfer@ihp-microelectronics.com (P.L.); 2 Brandenburg University of Technology Cottbus-Senftenberg, 03046 Cottbus, Germany

**Keywords:** elliptic curve cryptography (ECC), Montgomery *kP*, atomic patterns, B-curves, P-curves, side-channel analysis (SCA) attacks, simple SCA attacks, address-bit SCA, horizontal attacks, power analysis, electromagnetic analysis

## Abstract

Elliptic curve cryptography (ECC) is one of the commonly used standard methods for encrypting and signing messages which is especially applicable to resource-constrained devices such as sensor nodes that are networked in the Internet of Things. The same holds true for wearable sensors. In these fields of application, confidentiality and data integrity are of utmost importance as human lives depend on them. In this paper, we discuss the resistance of our fast dual-field ECDSA accelerator against side-channel analysis attacks. We present our implementation of a design supporting four different NIST elliptic curves to allow the reader to understand the discussion of the resistance aspects. For two different target platforms—ASIC and FPGA—we show that the application of atomic patterns, which is considered to ensure resistance against simple side-channel analysis attacks in the literature, is not sufficient to prevent either simple SCA or horizontal address-bit DPA attacks. We also evaluated an approach which is based on the activity of the field multiplier to increase the inherent resistance of the design against attacks performed.

## 1. Introduction

Elliptic curve cryptography (ECC) is an asymmetric cryptographic approach proposed independently in 1985 by Neal Koblitz [1] and Victor S. Miller [2]. Elliptic curves are used in various fields of application. Many interesting details about ECC including its genesis can be found in [3]. The advantage of ECC in comparison to RSA [4] is the possibility to use significantly shorter keys than RSA by maintaining the same security strength. ECC is used to share secret keys, for signing messages and for authentication, which are essential when it comes to communication, e.g., in the fields of the Internet of Things and wearable sensors [5]. The main operation of ECC is the elliptic curve point multiplication *k**P***, where ***P*** is a point of an elliptic curve (EC), and *k* is a scalar, also denoted as *key*. The length of the scalar depends on the type of EC used and on the security requirements. Currently, keys have to be about 200–300-bit long. There are two types of ECs standardized for use in cryptographic protocols: ECs over extended binary fields *GF*(2*^n^*), and ECs over prime fields *GF*(*p*). The *k**P*** operation is the most time- and energy-consuming operation in ECC protocols. The Montgomery *k**P*** algorithm (the *Montgomery ladder*) using Lopez–Dahab projective coordinates [6] is the algorithm most often used in hardware implementations for accelerating cryptographic operations for ECs over extended binary fields *GF*(2*^n^*) due to its fast execution time. It requires only six field multiplications for processing a key bit, whereby all other operations—field additions and squaring operations as well as register operations—can be implemented in parallel to field multiplications. For ECs over *GF*(*p*), no comparably fast algorithm is known. Usually, many more field multiplications have to be calculated. Additionally, mathematical operations—multiplications, additions and subtractions in prime fields—implemented in hardware are significantly slower than the mathematical operations in extended binary fields due to the delay caused by the carry bit propagation. For large networks or time-critical applications, a fast execution of cryptographic operations is essential. For automotive applications, at least 1000 signature verifications per second are required; in big cities, this number even reaches up to 4000. Corresponding to the Elliptic Curve Digital Signature Algorithm (ECDSA) [7], only one *k**P*** operation has to be calculated for a generation of a digital signature, but two *k**P*** operations have to be calculated for its verification. To fulfil the speed requirements, efficient methods have to be applied at each implementation level, i.e., field operations, *k**P*** operation and ECDSA acceleration. For example, the Straus–Shamir trick [8,9] is a well-known means to accelerate the signature verification for ECs over *GF*(*p*).

An additional requirement for applications that are sensitive to manipulations is that the implemented algorithms have to be resistant to side-channel analysis (SCA) attacks, if an attacker has physical access to the working cryptographic device. The goal of attackers is to reveal the scalar *k* processed during a *k**P*** execution. Many *k**P*** algorithms process the scalar *k* bitwise, i.e., *bit-by-bit*. The Montgomery *k**P*** algorithm for ECs over *GF*(2*^n^*) is known in the literature to be resistant against simple SCA attacks due to its regularity [10,11], i.e., the sequence of operations for the processing of the bit value “0” of the scalar *k* is the same as for the processing of the bit value “1”. Due to the fact that a point doubling and a point addition have to be calculated for processing each key bit in the main loop of the Montgomery ladder, the Montgomery *k**P*** algorithm can be considered a type of a *double-and-add-always* algorithm. In order to reduce the execution time of cryptographic operations, *k**P*** algorithms for ECs over *GF*(*p*) are often based on the binary *double-and-add* algorithm. In that case, the processing of the key bit “0” requires only a single point doubling calculation, but the processing of the key bit “1” requires two point operations—a point doubling and a point addition. The sequence of mathematical operations for a point doubling differs from that of a point addition. This results in a different energy consumption for each type of point operation, i.e., the power profile of a point doubling is distinguishable from the power profile of a point addition. If an attacker can measure the current drawn by the device or its electromagnetic emanation during a *k**P*** execution (hereafter *k**P*** trace), and the differences are observable by a visual inspection of the trace, the implementation is vulnerable to simple SCA attacks. To make the *double-and-add* algorithm resistant to simple SCA attacks, the sequence of the field operations for an EC point doubling has to be the same as the sequence of the field operations for an EC point addition: for example, using a unified point addition formula. Another strategy is the implementation of both point operations using a repeated (short) sequence of field operations. For example, a point doubling can be implemented as a sequence repeated 16 times containing “two additions, one additive inverse, one multiplication and four write-to-register operations”, and a point addition can be implemented as a sequence of the same operations repeated 10 times. This strategy was proposed in [12] and is known as the “atomicity” principle because the short operation sequence is denoted as an “atom”. In [13], longer atomic patterns were proposed for EC point doubling and addition, using special mixed Jacobian–affine coordinates. The point doubling as well as the point addition requires only 10 field multiplications, 10 field additions/subtractions and 21 write-to-register operations, i.e., this algorithm is also the fastest one (see Section 4 in [13]). 

This paper is an extended version of the paper entitled “Fast Dual-Field ECDSA Accelerator with Increased Resistance against Horizontal SCA Attacks” presented in 2021 at the IEEE International Conference on Cyber Security and Resilience (CSR) [14]. We implemented an elliptic curve point multiplication for the following four NIST elliptic curves [7]: *B-233*, *B-283*, *P-224* and *P-256*, in hardware to accelerate the generation and verification of digital signatures. The following contributions are described in [14] and here:

- Applying the four-segment iterative Karatsuba multiplication formula (MF) to both types of Galois fields. The multiplication formula was proposed and efficiently applied in [15] for accelerating the field multiplication in *GF*(2*^n^*). To the best of our knowledge, we are the first team to apply this formula to implement a dual-field multiplier. In comparison to the classical multiplication formula, the segmentation of multiplicands in four parts, the four-segment Karatsuba MF, is much faster: the product can be calculated in only nine clock cycles, which is seven clock cycles less than when applying the classical MF. It reduces the calculation time as well as the energy consumption of *k**P*** executions for ECs over *GF*(*p*) by about 40%. Thus, the automotive requirement to be able to perform 1000 signature verifications per second can be reached even with non-scaled technologies: for example, using a 250 nm gate library [16].
- Evaluation of the resistance of the implemented four-EC design against horizontal *address-bit* differential power analysis (DPA) attacks using the *comparison to the mean* method [17]. Analyzing a *k**P*** power trace simulated for EC *B-233* in the four-elliptic-curve design, we obtained only the two best key candidates with a correctness of about 87% and 85%, respectively. In comparison to this, attacking the single *B-233* curve *k**P*** design resulted in six key candidates being obtained, with a correctness higher than 95%—so the resistance of the four-elliptic-curve design is significantly better than that of the single designs.
- Verification of our assumption about the vulnerability of the atomic pattern algorithm [13] to horizontal SCA attacks. Analyzing power traces for EC *P-224*, we obtained four key candidates with a correctness of 97% or higher. Attacking EC *P-256*, we were able to reveal the key completely, i.e., three key candidates had a correctness of 100%.

For our implementation, we selected *k**P*** algorithms that are fast and mentioned in the literature as resistant against simple SCA attacks, due to the regularity and atomicity principles of the algorithms. More specifically, we selected the Montgomery ladder using projective Lopez–Dahab coordinates [6] for ECs over *GF*(2*^n^*), and the atomic pattern algorithm using mixed Jacobian–affine coordinates [13] for ECs over *GF*(*p*). 

The vulnerability of the Montgomery ladder to horizontal address-bit DPA attacks is known [18]. The key-dependent addressing of registers or other blocks is “visible” even through simple statistical analysis of a single *k**P*** trace. The resistance of the atomic pattern algorithm [13] against horizontal address-bit DPA attacks has, to the best of our knowledge, not been evaluated yet. We assumed that the power profiles of point doublings and point additions, implemented corresponding to the atomic patterns described in [13], are distinguishable due to the key-dependent use of registers in these patterns. Thus, the algorithm [13] is, theoretically, vulnerable to horizontal address-bit DPA attacks, too. However, we expected that the resistance of our design for four ECs will be higher than that of a design for a single EC, due to the fact that some operations (for example, field reductions) are always performed for all four ECs in our design. This increases the energy consumption of the *k**P*** design, but it is a type of noise that can hide the key-dependent addressing of the registers. Our assumptions were confirmed.

Additionally, we extended our conference paper [14] with the following investigations:

1. We extended the evaluation of our design’s resistance against horizontal DPA attacks. We ported the design to an FPGA, captured electromagnetic traces of the *k**P*** execution, repeated our attack analyzing the measured traces and compared the attack results with those obtained by attacking the simulated traces of an ASIC. Despite the significant differences between the target platforms—a cryptographic ASIC in the IHP 250 nm technology and an FPGA in the 28 nm technology—the resistance of the cryptographic designs against horizontal DPA attacks is quite similar, i.e., the performed attack was successful.
2. We performed additional investigations by applying an automated simple analysis attack against the measured electromagnetic traces for the ECs *P-224* and *P-256*. We discovered and demonstrated that the atomic patterns presented in [13] are vulnerable to simple analysis attacks.
3. We proposed an approach to reduce the success of simple analysis attacks, as well as horizontal attacks, and evaluated its effectiveness by attacking the electromagnetic traces of the modified design.

The rest of this paper is structured as follows. In Section 2, we focus on the implementation details of our unified design. The results of the horizontal DPA attacks performed against simulated power traces are presented and discussed in Section 3. Section 4 provides the results of a horizontal differential electromagnetic analysis performed against electromagnetic traces measured on an FPGA. In Section 5, we present the results of our automated simple analysis attack. An approach focused on reducing the success of horizontal attacks is presented and evaluated in Section 6. This paper ends with short conclusions.

## 2. Implementation Details

### 2.1. Accelerated Operations

In our hardware–software co-design for signature generation and verification corresponding to the ECDSA, we implemented elliptic curve point operations in hardware. We selected the following algorithms for the implementation:

- The modified Montgomery *k**P*** algorithm using Lopez–Dahab projective coordinates as described in [19] for the acceleration of the *k**P*** operations using the ECs *B-233* and *B-283*;
- The *double-and-add k**P*** algorithm with atomic patterns using mixed Jacobian–affine coordinates for point doublings and point additions (see patterns reported in [13], Section 4.1) as the basis for our accelerator for the ECs *P-224* and *P-256*. We applied the Straus–Shamir trick [8,9] as recommended in [13] for the calculation of *u**P** + v**Q***. We slightly modified the sequence of operations presented in [13] with the goal to increase the pipelining of field operations.

Our *k**P*** implementation is a bitwise processing of the scalar *k*. The Straus–Shamir trick (see Algorithm A1 in Appendix A) allows for performing only one *k**P*** operation instead of two independent elliptic curve point multiplications, *u**P*** and *v**Q***. The number of point doublings is unchanged, but the number of point additions is increased. This trick reduces the execution time of the (*u**P** + v**Q***) calculation significantly.

Pipelining of the field operations in our implementation as well as the applied four-segment Karatsuba multiplication formula for realizing the dual-field multiplier reduces the execution time of each *k**P*** operation by about 40% compared to the classical multiplication method. The time reduction has a similar effect to applying the Straus–Shamir trick.

The algorithms selected for the *k**P*** implementation and the four-segment Karatsuba multiplication formula for both Galois fields are presented in Appendix A (see Algorithm A2 and Equation (A1)) to increase the readability of this paper. In the rest of this section, we present the implemented sequence of the field multiplications and explain the structure of our design and its blocks.

### 2.2. Implemented Sequences of Operations

Table 1 shows the operation sequence we implemented in the main loop of our *k**P*** design for ECs over *GF*(2*^n^*), corresponding to Algorithm A2 in Appendix A. In the main loop, a single key bit value *k_i_* of the scalar *k* is processed.

In our implementation, the main loop consists of a sequence of six field multiplications (denoted *M*1–*M*6), five field squaring operations (denoted *Sq*1–*Sq*5), three field additions (denoted *Add*1, *Add*2, *Add*3) and many *write-to-register* operations (denoted with “←”). The field addition is a bitwise XOR operation. The field squaring of an element $A(t)=a_{n-1}t^{n-1}+a_{n-2}t^{n-2}+\ldots +a_{1}t+a_{0}$ ∈ *GF*(2*^n^*) with an irreducible polynomial *f*(*t*) can be performed as a multiplication with the same operands ${\left(A(t)\right)}^{2}=A(t)\cdot A(t)\;\mathrm{mod}f(t)$ or, alternatively, corresponding to the following formula using the binary representation of $A(t)=a_{n-1}a_{n-2}\ldots a_{0}=\sum_{i=0}^{n-1}a_{0}\cdot 2^{i}$:

(1) $${\left(A(t)\right)}^{2}=\left(\sum_{i=0}^{n-1}a_{i}\cdot 2^{2i}\right)\mathrm{mod}f(t)=a_{n-1}0a_{n-2}0\ldots 0a_{1}0a_{0}\;\mathrm{mod}f(t)$$

The squaring corresponding to (1) is an easy operation compared to a multiplication with the same operands. It requires only a single clock cycle, consumes much less energy and can be performed in parallel to other multiplications, accelerating the *k**P*** executions significantly. We realized the operation of the main loop using a field multiplier block, a field adder, a field “squarer” and only seven registers: *X_1_*, *X_2_*, *Z_1_*, *Z_2_*, *X_3_*, *X_4_* and *x*. The register x contains the x-coordinate of the input point ***P***. Please note that *a* and *m* in Table 1 are not physical registers. They are only variables that we use here to denote some intermediate values. In our implementation, all squaring operations, additions and write-to-register operations are realized in parallel to field multiplications. The operations performed in parallel are shown in the same cell in Table 1: for example, the squaring operations *Sq*1 and *Sq*2 and the multiplication *M*1. 

The first field multiplication *M*1 in the current loop iteration depends on the key bit value that was processed in the previous iteration, i.e., it is either *X_1_∙Z_2_* or *X_2_∙Z_1_* in our design. This key-dependent selection of the operands for the first multiplication in the loop was originally introduced in [20] in 2008. Using this type of multiplication swap, the multiplier can start the calculation of the new product directly after the calculation of *M*6 because it does not need to wait for the new operands. Please note that the multiplier consumes much less energy during idle clock cycles. In such a case, the energy consumption of other blocks, for example, an adder performing addition *Add*3 and storing its results in the register, can be observed better. Using the multiplication swap, it is possible to perform addition *Add*3 and store its result in a register fully parallel to the last multiplication, *M*6. Thus, this parallelization not only reduces the execution time of the main loop but also increases the resistance of the implementation against SCA attacks. 

Table 2 shows the operation sequence we implemented in our *k**P*** design for ECs over *GF*(*p*) based on Algorithm A3 in Appendix A. The processing of the key bit value *k_i_* = 0 requires only a single point doubling. The processing of the key bit value *k_i_* = 1 requires two point operations—a point doubling and a point addition. In Algorithm A3, a subtraction is performed after the first field multiplication, and after these two operations, an addition of the field elements is executed. In our implementation, we performed the addition before the subtraction, in parallel to the first multiplication. Operations performed in parallel are shown in the same row in Table 2. 

The field operations in *GF*(*p*) require more gates when implemented in hardware and are slow compared to operations in *GF*(2*^n^*). This is due to the carry bit propagation and the more complex reduction. We implemented squaring operations as multiplications with the same operands. The sequence of operations in our design for point doublings as well as for point additions consists of 10 field multiplications, 10 additions (subtractions) and write-to-register operations. Thus, we realized the operation flow presented in Table 2 using a field multiplier block, a field adder and 12 registers: *X_1_*, *X_2_*, *X_3_*, *Z_1_*, *Z_2_*; *X*, *Y*, *Z_q_*; and *R_0_*, *R_1_*, *R_2_*, *R_3_*. The registers *X* and *Y* contain the coordinates of the input point ***P*** and will be not written but only read during the point doublings as well as during the point additions.

### 2.3. Structure of Our Design

The operations listed in Table 1 and Table 2 define the structure of our *k**P*** design. It consists of:

- A dual-field multiplier, denoted as **MULT**, that can calculate a field product for each of the four selected ECs, i.e., *B-233*, *B-283*, *P-224* and *P-256*;
- An **ALU** block for addition/subtraction in both *GF*(*p*) fields as well as for addition and squaring operations in both *GF*(2*^n^*) fields;
- **Registers** for storing the inputs, outputs and intermediate data;
- A **Controller** that manages the data flow between the design components and defines which operation has to be performed in the current clock cycle, including storing the data in registers as well as reading the data from the registers, ALU and multiplier;
- A **BUS** that implements the data exchange between the blocks and the registers corresponding to the Controller signals.

**Figure 1:**
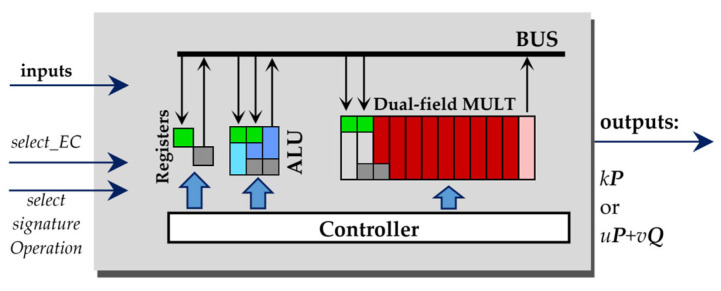
Structure of our design.

The structure of our design is illustrated in Figure 1.

The BUS is implemented as a multiplexer consisting of many logic gates that react on the address given by the Controller. All blocks including the registers can write their outputs to the BUS. Only one of the blocks can write its output data to the BUS in a certain clock cycle, but many blocks can read data from the bus at the same time. The *write-to-BUS* operation from the Controller connects the output of the source block to the inputs of all other blocks. By the *read-from-BUS* operation, only the destination block accepts the values on its input as data for processing. 

Reading data from the BUS and storing the data in a register require two clock cycles to be performed:

- In the first clock cycle, the data are written to the BUS, whereby the selected register (or the other selected design block) obtains the signal *we* (word enable) from the Controller. The green rectangle in Figure 1 indicates the addressing of the registers, ALU and MULT blocks.
- In the second clock cycle, the data are stored in the previously addressed register or in an internal register of a previously addressed block; data storing is denoted with a small gray rectangle in Figure 1.

The block ALU can perform different operations depending on the Controller signals as follows:

- Reading data from the BUS (green rectangle); the data will be stored in the internal register (gray rectangle in the block ALU).
- Squaring of *GF*(2*^n^*) elements (marked by yellow rectangles).
- Addition of field elements (marked by dark blue rectangles) in prime or binary extended fields.
- Subtraction of *GF*(*p*) elements (marked by magenta rectangles).
- Writing data to the BUS.

The block MULT performs a field multiplication for one of the four ECs. Depending on the signals from the Controller, the MULT block:

- Obtains two multiplicands: reading data from the BUS (see the two green rectangles) and storing the operand values in an internal register (see the two gray rectangles in block MULT). This requires two clock cycles.
- Calculates partial products according to the four-segment Karatsuba multiplication formula (see Equation (A1) in Appendix A), accumulates the partial products in its internal register and performs a field reduction. In each clock cycle, a single partial product will be calculated. Corresponding clock cycles are marked with a red rectangle. The reduction in the intermediate product value is also performed in each clock cycle, after the accumulation of the currently calculated partial product. This increases the energy consumption of the field multiplier slightly but also partially hides the key-dependent activity/addressing of other design blocks. Due to the applied four-segment Karatsuba multiplication formula, nine different partial products have to be calculated, i.e., the field multiplier needs nine clock cycles for the accumulation of a field product.
- Writes the calculated field product to the BUS directly after the nine partial products are accumulated.
- Stalls, i.e., calculates a partial product of two zero operands. During the calculation of the first such partial product directly after the processing of non-zero operands, the multiplier consumes a slightly reduced energy (this clock cycle is marked by light red). The energy consumption of the multiplier is small while stalling. We marked such clock cycles by the white color.

We implemented the partial multiplier in our MULT block using the classical multiplication formula, with the goal to reach the maximal operation frequency.

**Figure 2:**
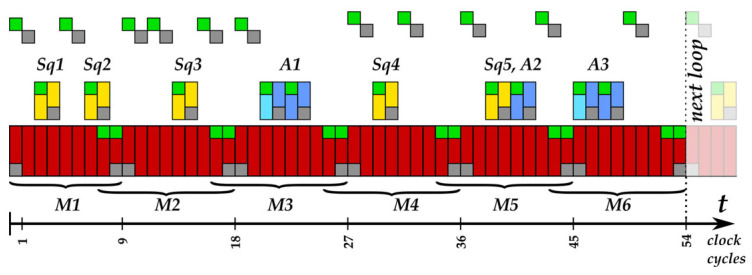
Processing sequence for a key bit in the main loop in our implementation for ECs over *GF*(2*^n^*), corresponding to Table 1.

Figure 2 shows the processing sequence in the main loop of our implementation for ECs over *GF*(2*^n^*), providing details about the activities of each block. Due to the structure of our design and the high level of pipelining achieved, the key bit processing requires only 54 clock cycles and is always performed using the same operation sequence, i.e., it is a regular processing of key bits. This operation sequence can be regarded as a type of “*atom*”, too. 

**Figure 3:**
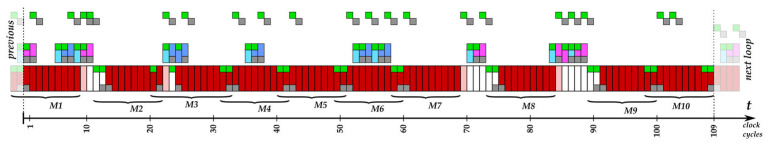
Operation sequence for a point doubling or a point addition in our implementation for ECs over *GF*(*p*), corresponding to Table 2.

Figure 3 shows the operation sequence for a point doubling and a point addition in our implementation for ECs over *GF*(*p*), providing details about the activities of each block.

For point additions using mixed Jacobian–affine coordinates [13], the atomic patterns do not allow such a high level of parallelization and pipelining. Even in our slightly modified sequence of operations, the addition *Add*3 and the subtractions *Add*2, *Add*7, *Add*8 and *Add*9 can be performed only between multiplications. Due to the realized pipelining and the fast field multiplier, a single atomic pattern—a point doubling or a point addition—requires only 109 clock cycles. Thus, the key bit value “0”, which requires only a point doubling, can be processed in 109 clock cycles. The processing of the key bit value “1” requires two atomic patterns (a point doubling and a point addition), i.e., it requires 2 × 109 = 218 clock cycles. Please note that a design that uses the classical multiplication method instead of the four-segment Karatsuba multiplication formula would require 179 clock cycles for an atomic pattern. Thus, the use of the four-segment Karatsuba multiplication formula reduces the execution time of a single atomic pattern by about 40%, whereby the maximal frequency, determined by the longest signal propagation path of the partial multiplier and the reduction unit, is the same.

In the next section, we explain how we performed horizontal DPA attacks. The details about the structure and the activity of the design blocks presented here are important for understanding the attack results and the reasons why the horizontal address-bit SCA attacks were successful.

## 3. Horizontal DPA Attack: Analysis of Simulated Traces

### 3.1. Power Trace Simulation

**Figure 4:**
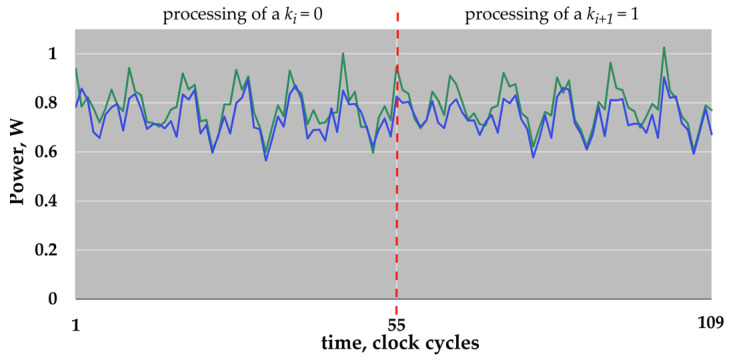
ECs *B-233* (blue line) and *B-283* (green line): a part of the simulated power traces of a *k**P*** execution corresponding to the processing of two key bits “01”.

We synthesized our ECDSA accelerator for the IHP 250 nm technology. We used the Very High Speed Integrated Circuit Hardware Description Language (VHDL) for the behavioral modeling of our design and synthesized it using the Synopsys Design Compiler Version K-2015.06-SP2. The maximum achieved clock frequency was 62.5 MHz (16 ns clock cycle period) after synthesis. For each of the four ECs, we generated power traces of a *k**P*** execution using Synopsys PrimePower Version Q-2019.12-SP1. The PrimePower tool uses the gate-level simulation activity stored in the activity file to estimate the averaged power consumption of the design. It can also precisely analyze the power consumption of the design for each clock cycle within a given time interval, i.e., it allows simulating a power trace of *k**P*** executions, with the given inputs. Power traces were simulated with a step of 0.01 ns, i.e., our trace contains 1600 simulated values per clock cycle. We do not need such a fine-grained simulation for the experiments described in this work. To simplify the analysis, and due to the fact that the simulated trace is noiseless, we performed a trace compression. For each clock cycle, we calculated the sum of its sampling values and represented it using this single value (i.e., the *y*-axis shows the average power consumption in W, multiplied by 1600). Figure 4 shows parts of the compressed power traces simulated for the ECs *B-233* and *B-283*. The parts shown correspond to the processing of two key bits, i.e., “01”.

**Figure 5:**
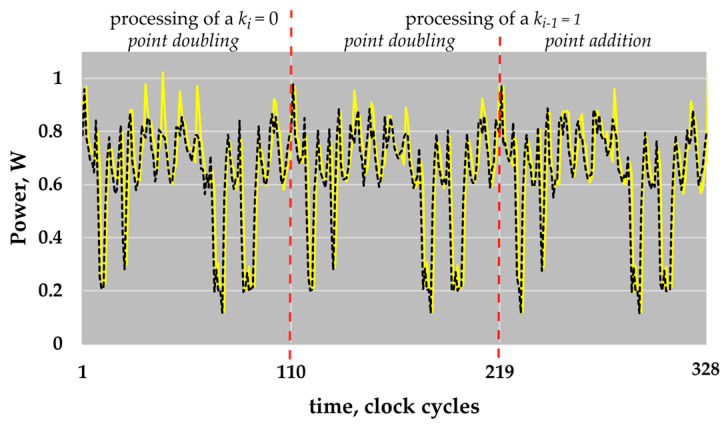
ECs *P-224* (black dotted line) and *P-256* (yellow line): a part of the simulated power traces of a *k**P*** execution corresponding to the processing of two key bits “01”.

Figure 5 shows parts of the compressed power traces simulated for the ECs *P-224* and *P-256*. The shown parts also correspond to the processing of two key bits “01”, i.e., here, three atomic patterns are shown: two point doublings, and a point addition. 

The clock cycles with reduced energy consumption are easy to see in Figure 5. As described above, the non-active field multiplier is the reason for the reduced energy consumption. These clock cycles are a type of marker. They “show” the periodicity of an atomic pattern. The knowledge about the period of atomic patterns is helpful for the preparation of different attacks. In comparison to the atomic patterns for the point operations in *GF*(*p*), the atomic patterns corresponding to the processing of a key bit in *GF*(2*^n^*) do not have such a type of marker in our implementation.

Please note that all traces shown in Figure 4 and Figure 5 are already compressed power traces, i.e., the row traces—the simulated power traces—were already partially prepared for the analysis. 

### 3.2. Horizontal Power Analysis Attack

In this paper, we concentrated on single-trace attacks that are also known as horizontal attacks. In our horizontal attacks, we did not exploit any key-dependent correlations based on the processing of the same/different data as in horizontal correlation analysis attacks [21], or the storing of data in registers as in template attacks [22]. Our attack did not exploit the key-dependent data flow. The attack was based on the assumption that key-dependent addressing of different registers or other blocks of a cryptographic design is distinguishable. This assumption was exploited in [23] in 2002 for revealing a key using 1000 *k**P*** traces, i.e., it was a successful vertical attack. Since 2017, successful horizontal attacks exploiting key-dependent addressing of the Montgomery ladder for ECs over *GF*(2*^n^*) have been reported [18]. In detail, the following points were assumed as the basis of these attacks:

- The attacked design processes a secret binary number *k* = *k*_*n*−1_*k*_*n*−2_…*k*_1_*k*_0_ bitwise;
- The addressing of the design blocks for processing the key bit value “0” differs from the addressing of the design blocks for processing the key bit value “1”;
- The key-dependent addressing of the design blocks makes the shapes of the processing of the key bit value “0” distinguishable from the shapes of the processing of the key bit value “1”;
- The differences in the shapes can be detected using statistical analysis methods or unsupervised machine learning algorithms.

Simple SCA attacks are successful if the power profiles of “0” key bits differ significantly from the power profiles of “1” key bits. These strong differences are often caused by the different sequences of mathematical operations in the main loop of the *k**P*** algorithm during the processing of “0” and “1” key bit values. These differences can be detected by visual inspection of the simulated or measured traces. Designs with the same sequence of operations for the processing of different key bit values—regular designs—are considered as resistant against simple SCA attacks. The differences caused by the key-dependent addressing of blocks are no longer significant, but they can still be “seen” using statistical analysis as a type of “magnifying glass”. Here, we performed an analysis using the *comparison to the mean* approach corresponding to [17]. 

In this work, we have two types of atomic patterns:

- Atomic patterns for a point doubling or a point addition for ECs over *GF*(*p*);
- Atomic patterns for the processing a key bit in the main loop of the *k**P*** algorithm for ECs over *GF*(2*^n^*).

Due to the duration of an atomic pattern (in clock cycles) and the compression of the power trace (only a single value represents each clock cycle), the power profile of an atomic pattern consists of:

- One hundred and nine sample values for ECs over *GF*(*p*);
- Fifty-four sample values for ECs over *GF*(2*^n^*).

We denote the current sample in an atomic pattern power profile (hereafter atomic profile) using the letter *j* in this work. Thus, for an atomic pattern:

- For ECs over *GF*(*p*), *j* ∈ {1, 2, …, 109};
- For ECs over *GF*(2*^n^*), *j* ∈ {1, 2, …, 54}.

We numbered each power profile of the processing of key bit *k_i_* in the main loop of the implemented algorithm using the same index letter *i.* Please note that in the main loop in our implementation for ECs over *GF*(2*^n^*), the key bits *k_l−_*_3_, *k_l−_*_4_,…, *k*_1_, *k*_0_ are processed, i.e., *i*∈{*l*−3, *l*−2,…, 1,0}, and for ECs over *GF*(*p*), *i*∈{*l*−2, *l*−1,…, 1,0}.

For the simulated, compressed *k**P*** traces of ECs *B-233* and *B-283*, we:

- Calculated the *mean atomic profile* averaging all atomic profiles.
- Compared the value of the sample number 1 in the mean atomic profile with the sample number 1 in the *i*th atomic pattern profile, for all *i* in the analyzed trace:
  ⚬ If the value of the sample number 1 in the *i*th atomic pattern profile was higher or equal to that in the *mean atomic profile,* we assumed that the key bit value *k_i_* = “1” was processed; otherwise, we assumed that the key bit value *k_i_* = “0” was processed.
  ⚬ Therefore, we made an assumption about all *l*−*2* bits of key candidate 1, i.e., we extracted *key_candidate_1*.
- Repeated the comparison for each of the remaining *j* sample numbers in order to extract the corresponding *key_candidate_j*.
- Evaluated the correctness of the extracted key candidates, i.e., we compared each *key_candidate_j* with the scalar *k* processed in the analyzed *k**P*** execution. The result of the comparison is the relative number of correctly revealed key bits. For example, if 200 bits of a key candidate were revealed correctly and the scalar *k* processed was 233-bit long, the correctness *δ* = (200/233)⸱100% ≈ 85.8%.

We analyzed the *k**P*** traces for ECs *P-224* and *P-256* in a similar way. We calculated the mean power profile of point operations performed in the main loop of the *k**P*** executions for ECs *P-224* and *P-256*. The mean profile contains 109 samples—one sample per clock cycle. We compared the mean profile of the point operation calculated for a *k**P*** execution with each point operation profile in the *k**P*** execution trace sample-wise. Using the mean atomic profile, we made an assumption about the point operations, i.e., we distinguished point additions and point doublings. Each point doubling profile followed by a point addition profile was denoted as a processed key bit value of “1”, corresponding to the implemented *double-and-add* algorithm. For the evaluation of each extracted key candidate, we calculated its correctness as described above. 

Thus, for each *k**P*** trace analyzed for ECs over *GF*(*p*), we extracted 109 key candidates, and for ECs over *GF*(2*^n^*), we extracted 54 key candidates.

Please note that if the correctness of a key candidate is less than 50%, it means that our assumption about the processed key bit value was wrong. Bitwise inversion of the key candidate gives us the key candidate, for which the correctness is (100% − *δ*) > 50%.

**Figure 6:**
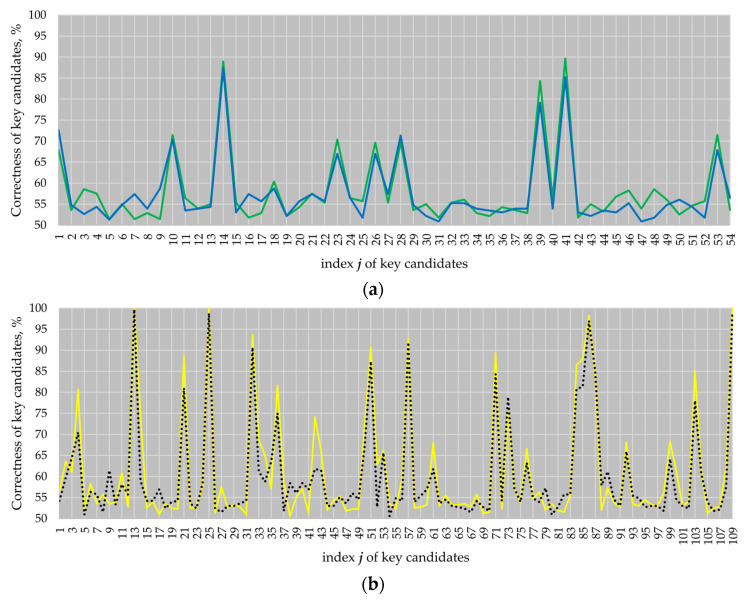
Attack results: (**a**) ECs *B-233* (blue line) and *B-283* (green line); (**b**) ECs *P-224* (black dotted line) and P-256 (yellow line).

Figure 6a shows the attack results for the ECs *B-233* and *B-283*, and Figure 6b shows the attack results for the ECs *P-224* and *P-256*. 

**Figure 7:**
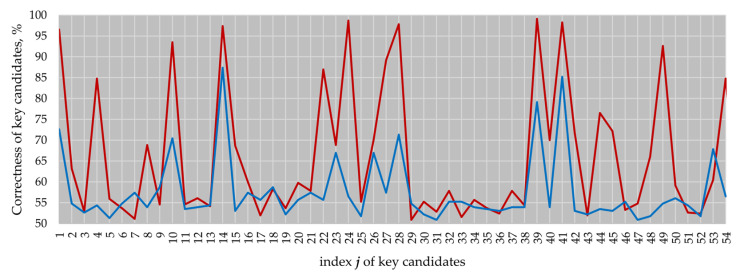
Attack results analyzing *k**P*** traces of EC *B-233*: the red line corresponds to the design implementing the *B-233* curve only; the blue line shows the resistance of our four-EC design described here, i.e., it corresponds to the blue line in Figure 6a.

The results of our attacks demonstrate that the atomicity patterns do not prevent horizontal attacks from being successful. On attacking EC *P-224*, we obtained four key candidates with a correctness higher than 97% (see key candidates 13, 25, 86 and 109). On attacking EC *P-256*, we fully revealed the key (see key candidates 13, 25 and 109 with a correctness of 100%). Well-known randomization techniques such as secret scalar randomization, randomization of the projective EC point coordinates or EC point blinding [24] are effective against vertical attacks, i.e., if key-dependent data processing is exploited to extract the key. However, they are not effective [25,26] against attacks exploiting the key-dependent addressing of the design blocks, i.e., their application when using atomic patterns does not prevent horizontal attacks that exploit the addressing from being successful. In other words, they do not prevent simple SCA attacks that are looking for highly fine-grained differences in the trace. The weak point of the atomicity principle is the assumption that the addressing of different registers (or other blocks of the design) is indistinguishable. The same weak point cryptographic designs have when implementing the regularity principle as a countermeasure against simple SCA attacks. This does not hold true if the design is a hardware implementation. Therefore, approaches for hiding the addressing have to be applied in addition. One of the well-known approaches is adding noise. The activity of design blocks is a type of noise. Figure 7 demonstrates how the activity of the blocks in our four-curve design decreased the success of the same attack performed analyzing a *k**P*** trace recorded when only the EC *B-233 k**P*** design was active. In the case of the single EC *B-233 k**P*** design, we obtained 12 key candidates with a correctness higher than 85%, whereby 6 of the 12 key candidates had a correctness higher than 95% (see red line in Figure 7). In the case of the four-active-EC design, only two key candidates had a correctness of about 85%, whereby the correctness of the best key candidate was 87% (see blue line in Figure 7).

## 4. Horizontal DPA Attack: Evaluation of an FPGA Implementation

**Figure 8:**
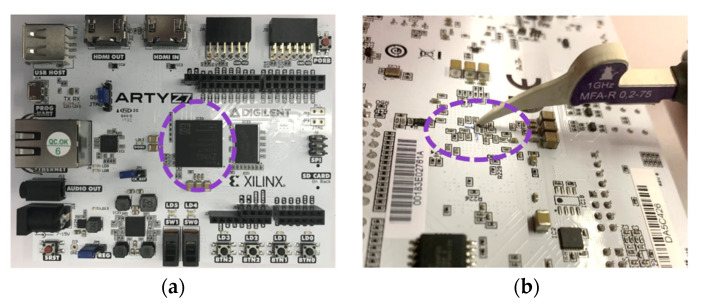
Arty Z7-20 development board (**a**), and the measurement location on its backside (**b**).

We ported our design to an FPGA with the goal to confirm the conducted investigations on a real device. As the target platform, we took an Arty Z7-20 development board (Figure 8a) equipped with a Xilinx 7-series FPGA produced in a 28 nm technology. The attacked FPGA is marked with a circle in Figure 8. 

Table 3 shows the FPGA resources used by the whole design as well as its multiplier block.

The maximum operating frequency for the design is slightly above 25 MHz. However, we applied a clock frequency of 10 MHz to increase the number of captured samples per clock cycle during the collection of *k**P*** traces.

The board used is not suitable for the connection of a current probe. Therefore, instead of measuring the power traces that require a board modification, we applied a near-field magnetic probe to capture electromagnetic traces. The electromagnetic traces were captured during the execution of *k**P*** operations at a 10 GS/s sampling rate by a LeCroy WavePro 254HD oscilloscope and a Langer MFA-R 0.2-75 [27] near-field probe connected to it. We placed the MFA-R probe close to one of the power decoupling capacitors. The exact location of the probe is shown in Figure 8b. 

**Figure 9:**
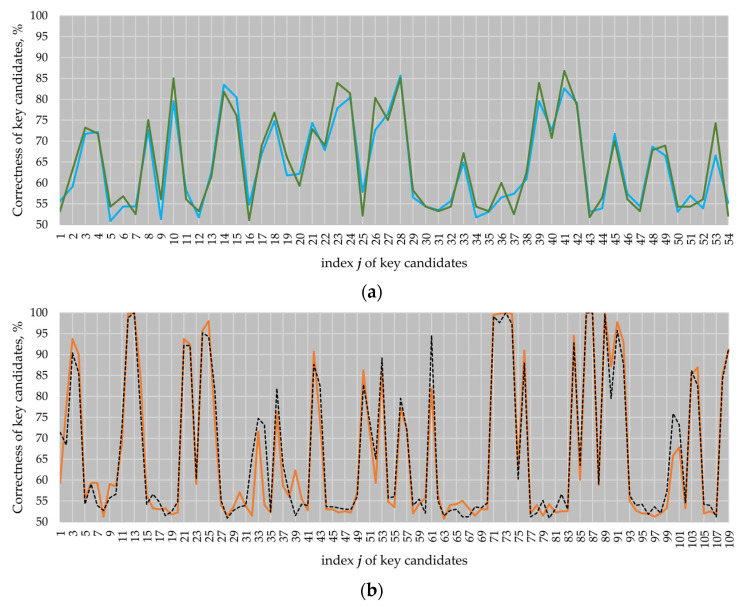
Attack results for the EM traces measured on an FPGA: (**a**) ECs *B-233* (blue line) and *B-283* (green line); (**b**) ECs *P-224* (black dotted line) and *P-256* (orange line).

We repeated our *comparison to the mean* attack against measured traces of the *k**P*** execution as described in Section 3. All the design inputs for the *k**P*** operations, i.e., coordinates of the EC points as well as scalars for each of the curves, were kept the same as those used during the simulations of the power traces. Figure 9a shows the attack results for the ECs *B-233* and *B-283*, and Figure 9b shows the attack results for the ECs *P-224* and *P-256*. 

On attacking EC *P-224*, we obtained four key candidates with a correctness of 100%, i.e., we fully revealed the key (see key candidates 13, 73, 86 and 87). On attacking EC *P-256*, we obtained five key candidates (clock cycles 13, 73, 86, 87 and 89) that are identical to the processed key.

Table 4 summarizes the results of the *comparison to the mean* attack performed against the simulated power traces and measured electromagnetic traces of the *k**P*** executions for the four investigated curves.

As it can be seen from Table 4, despite the significant difference between the target platforms used for the simulation and measurements of the traces (i.e., ASIC and FPGA), the results are quite similar. For the binary elliptic curves investigated here, there were no key candidates revealed with a correctness of more than 90% using the traces simulated for the ASIC as well as using the traces measured on the FPGA. In contrast to this, for the investigated elliptic curves over prime fields, the results obtained using the measured traces are far worse than for the simulated traces. The number of key candidates extracted with a correctness of more than 90% increased from 6 and 7 for ECs *P-224* and *P-256*, respectively, to 18 and 20.

## 5. Automated Simple Analysis Attack

We decided to investigate our design further by analyzing the uncompressed measured traces of the elliptic curves *P-224* and *P-256*. Analysis of uncompressed traces can provide advantages to designers for determining the leakage sources as well as attackers when revealing the key. If the leakage source is not very high/strong, or if the leakage duration is short in comparison to that of the clock cycle, the compression can “hide” this leakage, at least partially. 

The main purpose of the atomicity principle is to protect elliptic curve scalar multiplication against simple side-channel analysis attacks. Therefore, we applied an automated simple analysis attack against measured traces as described in [28], i.e., we tried to distinguish the point doubling atomic pattern from that of the point addition using our software that helps to detect tiny differences in a similar way to a magnifying glass.

As already mentioned before, the design was run at a 10 MHz clock frequency, and the traces were captured by an oscilloscope at a 10 GS/s sampling rate. These parameters resulted in 1000 measured samples per clock cycle. Hence, a single atomic pattern, consisting of 109 clock cycles in our implementation, is represented by 109,000 values within the measured trace.

**Figure 10:**
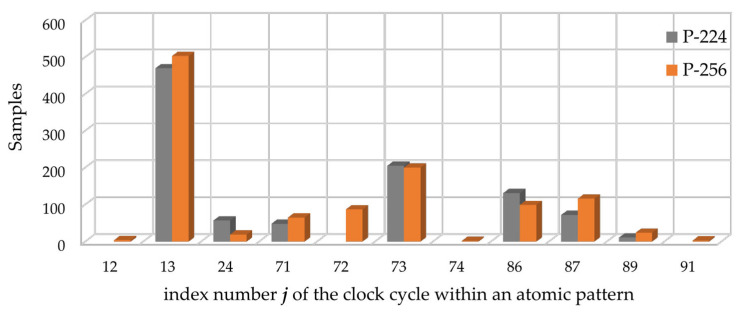
Visualization of the data from Table 5. Gray and orange bars correspond to the data obtained analyzing a *k**P*** trace for the ECs *P-224* and *P-256*, respectively.

In total, for each of the traces, out of 109,000 samples in the atomic pattern, we identified 994 samples for EC *P-224* and 1122 samples for EC *P-256* that allow for successfully revealing the processed scalar using simple visual inspection. Detailed information about the distribution of such samples within the atomic pattern as well as visualization of such data is presented in Table 5 and Figure 10.

Taking into account the information presented in Figure 3 and Figure 10, it is obvious that all the significant leakage comes from the clock cycles of the atomic pattern in which the multiplier block is not active. The multiplier’s inactivity results in a significant reduction in the energy consumed. Thus, the “contribution” of the other blocks to the energy consumption of the whole design becomes more “visible”. 

**Figure 11:**
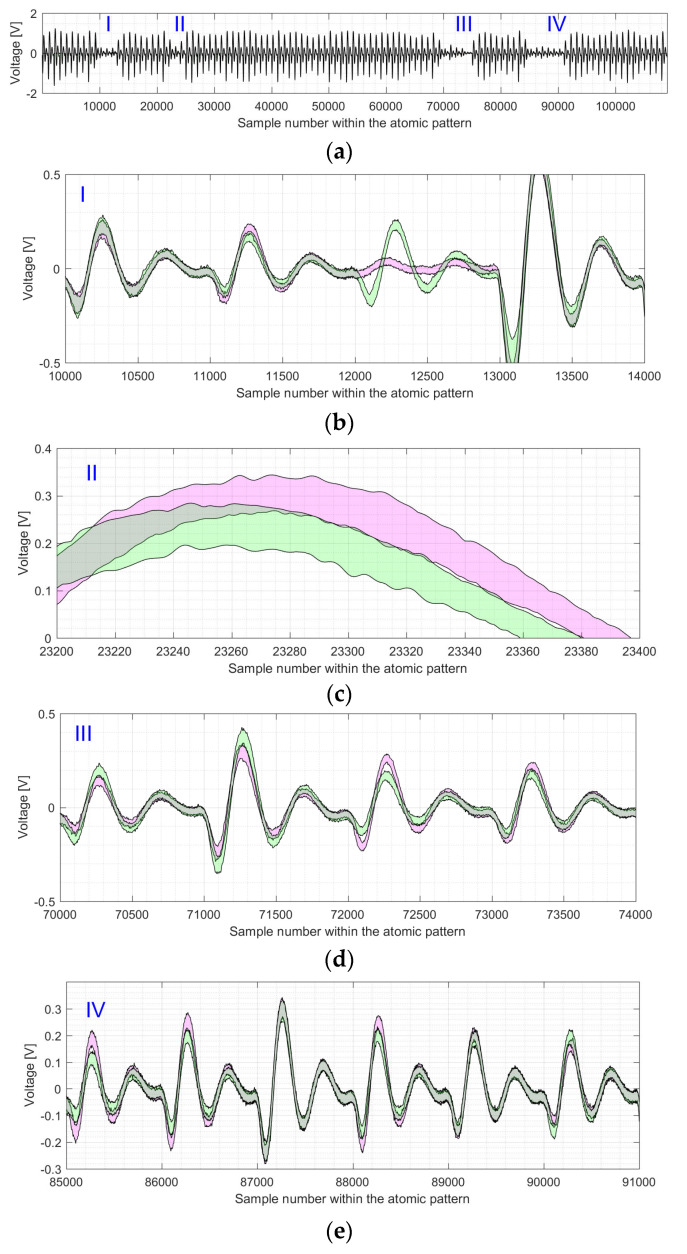
Shapes for the processing of EC point doublings (see green marked area) and EC point additions (see magenta marked area) for the *P-224* elliptic curve: (**a**) shows the shapes of all atomic patterns with 4 regions (zoomed in on (**b**–**e**)) where the multiplier is not active; (**b**) shows the region that corresponds to clock cycles 11 to 14; (**c**) shows a part of the 24th clock cycle; (**d**) shows clock cycles 71 to 74 of the atomic pattern; (**e**) shows clock cycles 86 to 91 of the atomic pattern.

We use an example of the electromagnetic trace for EC *P-224* to visualize the difference between the shapes of the point doubling and point addition atomic patterns, indicated by I–IV (see Figure 11a). Parts I–IV are depicted—zoomed in—in Figure 11b–e. The shapes of point additions and point doublings are represented by magenta and green areas, respectively.

**Figure 12:**
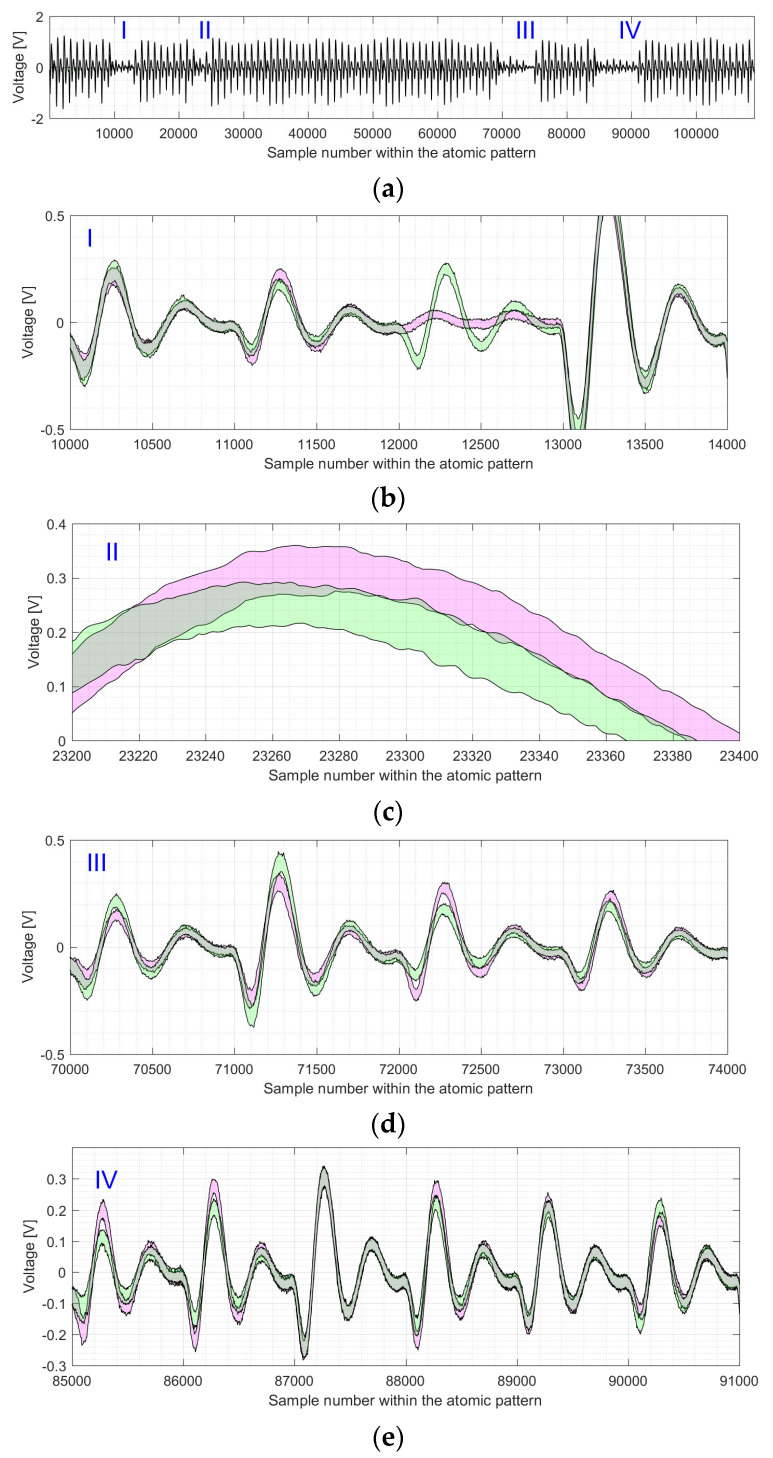
Shapes for the processing of EC point doublings (see green marked area) and EC point additions (see magenta marked area) for the *P-256* elliptic curve: (**a**) shows the shapes of all atomic patterns with 4 regions (zoomed in on (**b**–**e**)) where the multiplier is not active; (**b**) shows the region that corresponds to clock cycles 11 to 14; (**c**) shows a part of the 24th clock cycle; (**d**) shows clock cycles 71 to 74 of the atomic pattern; (**e**) shows clock cycles 86 to 91 of the atomic pattern.

As it can be seen in Figure 11 and Figure 12, the four regions with an inactive multiplier differ significantly in the level of the SCA leakage. Please note that the figures have different y-axis scales. The most “visible” leakage is located in the 13th clock cycle of the atomic pattern (see Figure 11b and Figure 12b). The green and magenta shapes are completely separated from one another in most of the points for the x-axis values in the range between 12,000 and 13,000. The leakage in clock cycle 24 is almost “invisible” as there is only a small separation for the x-range between 23,330 and 23,380 in Figure 11c and close to the sample number 23,320 in Figure 12c. From the designers’ point of view, the best case in terms of resistance against simple analysis attacks corresponds to the completely gray areas (i.e., the overlapping one), where the operation execution profiles cannot be distinguished from each other. 

The multiplier is a big block of our *k**P*** design. As it can be seen from Table 3, it consumes all the DSP blocks, almost half of the LUTs (49.94%) and 21.92% of flip-flops required by the whole design. Its energy consumption is high in comparison to other design blocks. Thus, the activity of the multiplier is a type of noise that can hide the activity of other blocks. In the next section, we demonstrate how the multiplier’s activity can reduce the success of horizontal attacks as well as simple analysis attacks as a particular case.

Figure 12 shows the difference between the shapes of atomic patterns, i.e., point doublings and point additions, for the electromagnetic trace of the EC *P-256 k**P*** execution.

## 6. Influence of Dummy Partial Multiplications on the Design Resistance

In our recent works [29,30,31], we investigated the influence of the activity of different field multipliers on the resistance of *k**P*** designs against horizontal SCA attacks. In [29], we investigated the application of the dummy activity of the multiplier as a means to reduce the vulnerability of our hardware accelerator for a single EC, *P-256*, to horizontal attacks. For this investigation, the design was derived from our unified four-curve ECDSA accelerator described here. Therefore, we assume that this approach should affect the unified design in a similar way. 

We modified our unified design by adding the execution of dummy partial multiplications in clock cycles where the multiplier block was not active before. The design was ported to the same FPGA, and the resource utilization is presented in Table 6. 

In comparison to the original design (see Table 3), the modification introduced an overhead of about 1.3% in terms of LUT utilization. The most affected block is the multiplier, which was increased by 459 LUTs.

**Figure 13:**
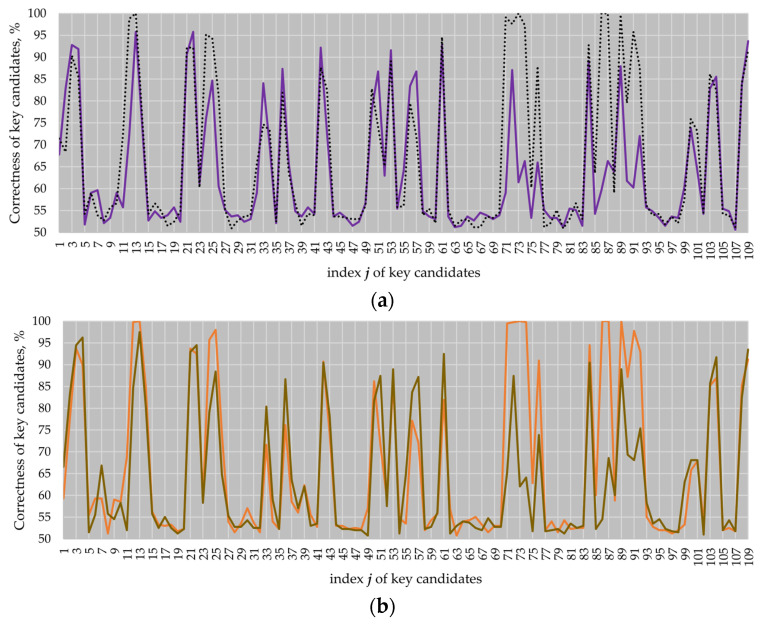
Attack results for the *k**P*** traces of ECs *P-224* (**a**) and *P-256* (**b**). Violet and brown lines correspond to the design with dummy partial multiplications; black dotted and orange lines correspond to the original design and are given as a reference.

We measured four electromagnetic *k**P*** traces on the modified design using the same inputs as in the previous experiments. We performed the *comparison to the mean* attack against each of the measured traces. For the *B-233* and *B-283* elliptic curves, the attack results are practically the same with a marginal difference. The attack results for the *P-224* and *P-256* elliptic curves are presented in Figure 13 and compared with the original design, i.e., the design without the dummy activity of the field multiplier. 

As it can be seen in Figure 13, the correctness of the key candidates for most of the clock cycles in the atomic pattern regions where the multiplier was not active, i.e., clock cycles 11 to 13, 24, 71 to 75 and 86 to 91 (see black dotted and orange lines), significantly dropped down (see violet and brown lines). 

The number of key candidates for EC *P-224* revealed with a correctness of more than 90% dropped down from 18 to 10 in comparison to the original design, while the best key candidate had a correctness of 97.49%. For EC *P-256*, the number of key candidates with a correctness of more than 90% dropped down from 20 to 9. The best key candidate had a correctness of 95.78%. Thus, the application of dummy partial multiplications reduced the success of the comparison to the mean attack.

Additionally, we repeated the simple analysis attack against the design with dummy partial multiplications using traces of the *P-224* and *P-256 k**P*** executions. As a result, we discovered 17 out of 109,000 samples that allow revealing the full key for the *P-224* trace only. All “leaky” samples were located in clock cycle 13 of the atomic pattern. 

The number of samples that allow revealing the full key decreased from 994 down to 17 samples for EC *P-224* and from 1122 to 0 samples for EC *P-256*. 

**Figure 14:**
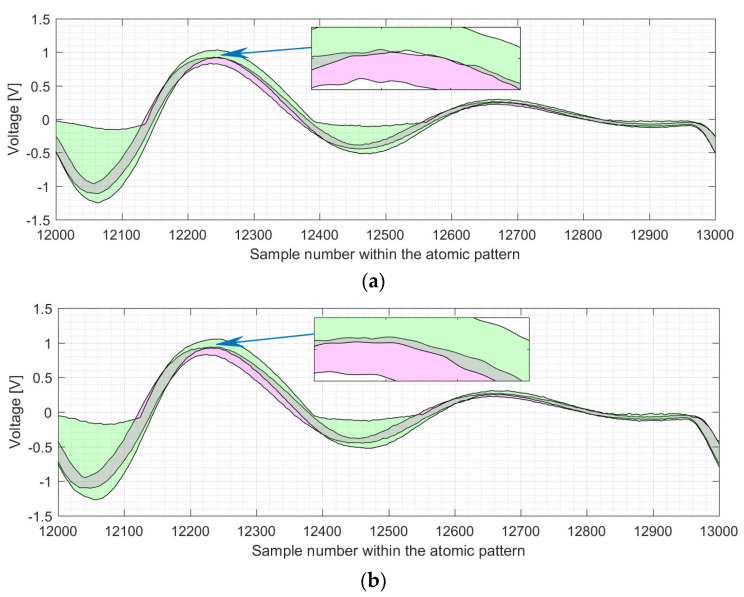
Shapes for the processing of EC point doublings (see green marked area) and EC point additions (see magenta marked area) in clock cycle 13 for *P-224* (**a**) and *P-256* (**b**).

The differences in the shapes of the point doublings and point additions are demonstrated in Figure 14a,b for clock cycle 13 of the atomic pattern for *P-224* and *P-256*, respectively.

## 7. Conclusions

In this paper, we focused on the side-channel analysis attack resistance of our design supporting four different elliptic curves, two *B*-curves over *GF*(2*^n^*) and two *P*-curves over *GF*(*p*). We used the four-segment Karatsuba multiplication formula for implementing a dual-field multiplier in our design, reducing the execution time of *k**P*** calculations by about 40% in comparison to the classical multiplication formula. Please note that the implementation of the *P*-curves employs atomic patterns that are considered as a means to render simple side-channel analysis attacks void. However, in this paper, we could show that the application of the atomic patterns introduced in [13] is not sufficient to prevent either simple SCA or horizontal address-bit DPA attacks, which are single-trace attacks. The source of the leakage is related to the key-dependent addressing of design blocks/registers in the atomic patterns, i.e., the assumption about the indistinguishability of the addressing of the design blocks/registers has to be revised. We could show that noise produced by the activity of the field multiplier can reduce the success of attacks applied and therefore increase the inherent resistance of the whole design. Designers have to be aware of such details and avoid a straightforward implementation of cryptographic algorithms that may result in a design vulnerable even to simple SCA attacks. 

## Figures and Tables

**Table 1:** Operation flow in the main loop iterations in our implementation for ECs over *GF*(2*^n^*).

Operation	*k_i_* = 0	*k_i_* = 1
*M*1	*Z_2_ ← X_1_∙Z_2_* (or *X_4_ ← X_2_∙Z_1_*)	*Z_1_ ← X_1_∙Z_2_* (or *X_4_ ← X_2_∙Z_1_*)
*Sq*1	*X_3_ ← Z_1_^2^*	*X_3_ ← Z_2_^2^*
*Sq*2	*a = Z_1_^4^*	*a = Z_2_^4^*
*M*2	*X_1_ ← X_2_∙Z_1_* (or *Z_2_ ← X_1_∙Z_2_*)	*X_2_ ←X_2_∙Z_1_* (or *Z_1_ ← X_1_∙Z_2_*)
	*Z_1_ ←a*	*Z_2_ ←a*
*Sq*3	*X_2_ ← X_1_^2^*	*X_1_ ← X_2_^2^*
*M*3	*X_1_ ← b∙Z_1_*	*X_2_ ← b∙Z_2_*
*Add*1	*a = Z_2_ + X_1_* (or *a = X_4_ + Z_2_*)	*a = Z_1_ + X_2_* (or *a = X_4_ + Z_1_*)
*M*4	*Z_1_← X_1_∙Z_2_* (or *Z_1_ ← X_4_∙Z_2_*)	*Z_2_ ← X_2_∙Z_1_* (or *Z_2_ ← X_4_∙Z_1_*)
*Sq*4	*Z_2_ ← a^2^*	*Z_1_ ← a^2^*
*M*5	*m = x∙Z_2_*	*m = x∙Z_1_*
*Sq*5, *Add*2	*X_1_ ← X_2_^2^ + X_1_*	*X_2_ ← X_1_^2^ + X_2_*
*M*6	*Z_1_ ← X_3_ ∙ X_2_*	*Z_2_ ← X_3_ ∙ X_1_*
*Add*3	*X_2_ ← m + Z_1_*	*X_1_ ← m + Z_2_*

**Table 2:** Operation flow for point doublings and point additions in our design for ECs over *GF*(*p*).

Title 1	Point Doubling: 2*S*	Point Addition: *S* + *P*
	inputs:	inputs:
	***S** =* (*X_1_:X_2_:X_3_:Z_1_:Z_2_*)	***S*** = (*X_1_*:*X_2_*:*X_3_*:*Z_1_*:*Z_2_*)
		***P*** = (*X*:*Y*:*Z_q_*)—input point
*M*1	*R_0_ ← X_3_ ∙ X_3_*	*R_1_ ←X ∙ Z_1_*
*Add*1	*R_2_ ← X_2_ + X_2_*	***R_2_ ← X_2_ + X_2_***
*Add*2	*R_1_ ← X_1_ − R_0_*	*R_1_ ← R_1_ − X_1_*
*M*2	*Z_1_ ← X_2_ ∙ R_2_*	*R_2_ ← R_1_ ∙ R_1_*
*Add*3	*X_2_ ← Z_1_ + Z_1_*	***R_0_ ← R_2_ + R_2_***
*M*3	*R_3_ ← R_2_ ∙ X_3_*	*R_3_ ← X_1_ ∙ R_2_*
*M*4	*R_2_ ←X_2_ ∙ X_1_*	*R_0_ ←Y ∙ Z_2_*
*Add*4	*X_1_ ← X_1_ + R_0_*	***Z_2_ ←Z_2_ + R_0_***
*M*5	*R_0_ ←R_1_ ∙ X_1_*	*Z_2_ ←R_1_ ∙ R_2_*
*M*6	*R_1_ ← Z_1_ ∙ X_2_*	*R_2_ ← X_3_ ∙ R_1_*
*Add*5	*X_1_ ← R_0_ + R_0_*	*X_1_ ←R_3_ + R_3_*
*Add*6	*R_0_ ← R_0_ + X_1_*	*X_1_ ← Z_2_ + X_1_*
*M*7	*X_1_ ←* (*R_0_*)*^2^*	*Z_1_ ←*(*X_1_*)*^2^*
*Add*7	*X_1_ ← X_1_ − R_2_*	*R_0_ ← R_0_ − X_2_*
*M*8	*Z_1_ ←* (*R_3_*)*^2^*	*R_1_ ←*(*R_0_*)*^2^*
*Add*8	*X_1_ ← X_1_ − R_2_*	*X_1_ ←R_1_ − X_1_*
*Add*9	*R_2_ ← R_2_ − X_1_*	*R_1_ ←R_3_ − X_1_*
*M*9	*Z_2_ ← Z_1_ ∙ R_3_*	*R_3_ ← R_1_ ∙ R_0_*
*M*10	*X_2_ ← R_0_ ∙ R_2_*	*R_0_ ← X_2_ ∙ Z_2_*
	*X_3_ ← R_3_*	*X_3_ ← R_2_*
*Add*10	*X_2_ ← X_2_ − R_1_*	*X_2_ ←R_3_ − R_0_*
	**output:**	**output:**
	*2**S** =* (*X_1_:X_2_:X_3_:Z_1_:Z_2_*)	***S** + **P** =* (*X_1_:X_2_:X_3_:Z_1_:Z_2_*)

**Table 3:** Arty Z7-20 FPGA resources used by the whole design and the multiplier block.

Resource	Available	Design Utilization	Multiplier Block Utilization
LUT	53,200	33,897 (63.72%)	16,928 (31.82%)
FF	106,400	11,150 (10.48%)	2445 (2.30%)
DSP	220	17 (7.73%)	17 (7.73%)

**Table 4:** Results of the *comparison to the mean* attack applied to simulated and measured traces.

Elliptic Curve	Number of Key Candidates Extracted with a Correctness *δ*			
	Simulated Traces		Measured Traces	
	80% ≤ *δ* < 90%	*δ* > 90%	80% ≤ *δ* < 90%	*δ* > 90%
*B-233*	2	0	5	0
*B-283*	3	0	8	0
*P-224*	6	6	12	18
*P-256*	8	7	9	20

**Table 5:** Distribution of the “leaky” samples over the atomic pattern.

**Clock Cycle**	12	13	24	71	72	73	74	86	87	89	91
	**Number of samples with a high leakage**										
**EC *P-224***	-	470	57	48	-	205	-	131	72	11	-
**EC *P-256***	4	503	19	65	87	200	2	99	116	24	3

**Table 6:** Arty Z7-20 FPGA resources used by the modified design and the multiplier block.

Resource	Available	Design Utilization	Multiplier Block Utilization
LUT	53,200	34,336 (64.54%)	17,387 (32.68%)
FF	106,400	11,149 (10.48%)	2445 (2.30%)
DSP	220	17 (7.73%)	17 (7.73%)

**Figure A1:**
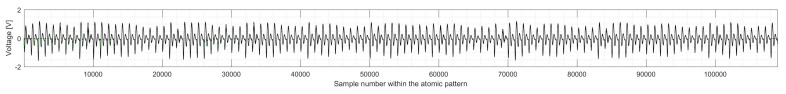
Atomic pattern shapes of the EMT trace for EC *P-224* with a design with dummy partial multiplications.

## Data Availability

Not applicable.

## Appendix A

**Algorithm A1**: Calculation of *u*∙***P*** + *v*·***Q*** using the Straus–Shamir trick.
Input: *u* = (*u_l−1_* … *u_1_ u_0_*)_2_, *v* = (*v_l−1_* … *v_1_ v_0_*)_2_ with (*u_l−1_*,*v_l−1_*) *≠* (*0*,*0*);
***P***, ***Q*** are points of EC over *GF*(*p*)*, **P ≠ Q***
Output: ***R*** = *u∙**P +** v·**Q***
Precompute: ***W** = **P** + **Q***
***R*** *= u_l−1_* *·**P**+ v_l−1_ ·**Q***
**for** *i* **from** *l − 2* **downto** *0* **do**
*R = 2·**R***
**if** *u_i_ = 1* **and** *v_i_ = 1* **then *R*** *= **R** + **W***
**else if** *u_i_ = 1* **and** *v_i_ = 0 **then R** = **R** + **P***
**else if** *u_i_ = 0* **and** *v_i_ = 1 **then R** = **R** + **Q***
**end for**
**return R**

**Algorithm A2**: Montgomery ladder using projective Lopez–Dahab coordinates.
Input: *k* = (*k_l−1_* … *k_1_ k_0_*)_2_ with *k_l−1_ = 1, **P** =* (*x,y*) *is a point of EC over GF*(2*^l^*)
Output: *k**P** =* (*x_1_, y_1_*)
initialization
**1**: *X_1_ ← x, X_2_ ← x^4^ + b, Z_2_ ← x^2^*
processing the second most significant bit
**2**: **if***k_l−2_* = 1 **then**
**3**: *T ← Z_2_, Z_1_ ←* (*X_1_Z_2_ + X_2_*)*^2^,*
*X_1_ ← X_1_Z_2_X_2_ + xZ_1_*,
**4**: *T ← X_2_, U ← b Z_2_^4^, X_2_ ← X_2_^4^+ U,*
*U ← TZ_2_, Z_2_ ← U ^2^.*
**5**: **else**
**6**: *T ← Z_2_, Z_2_ ←* (*X_1_Z_2_ + X_2_*)*^2^,*
*X_2_ ← X_1_X_2_T + xZ_2_,*
**7**: *T ← X_1_, U ← bX_2_^4^, X_1_ ← X_1_^4^ + b,*
*U ← TX_2_, Z_1_ ← T^2^.*
**8**: **end if**
main loop
**9**: **for***i***from***l − 3***downto***0***do**
**10**: **if***k_i_ = 1***then**
**11**: *T ← Z_1_, Z_1_ ←* (*X_1_Z_2_ + X_2_Z_1_*)*^2^, X_1_ ← xZ_1_ + X_1_X_2_TZ_2_,*
**12**: *T ← X_2_, X_2_ ← X_2_^4^ + bZ_2_^4^, Z_2_ ← T^2^ Z_2_^2^.*
**13**: **else**
**14**: *T ← Z_2_, Z_2_ ←* (*X_2_Z_1_ + X_1_Z_2_*)*^2^, X_2_ ← xZ_2_ + X_1_X_2_TZ_1_,*
***15:*** *T ← X_1_, X_1_ ← X_1_^4^ + b Z_1_^4^, Z_1_ ← T^2^ Z_1_^2^.*
**16**: **end if**
**17**: **end for**
end of the main loop
calculating affine coordinates of the *k**P*** result
**18**: *x_1_ ← 1/*(*xZ_1_Z_2_*)
**19**: *y_1_ ← y +* (*x + x_1_*)[(*X_1_ + xZ_1_*)(*X_2_ + xZ_2_*) *+* (*x^2^ + y*)(*Z_1_Z_2_*)] ∙ *x_1_*
**20**: *x_1_ ← X_1_x_1_xZ_2_//*i.e., *x_1_ = X_1_/Z_1_*
**21: return** (*x_1_*, *y_1_*)

**Algorithm A3:***Atomic patterns* using special mixed Jacobian–affine coordinates for EC point addition and doubling for ECs over *GF*(*p*) with the parameter *a = −3*, corresponding to [13].		
**Nr**	**point addition**	**point doubling**
1	*R_1_ ←X_q_ ∙ Z^2^*	*R_0_ ← I ∙ Z^2^*
2	*R_1_ ← R_1_ − X*	*R_1_ ← X − R_0_*
3	*⁕* *←**⁕* *+**⁕*	*R_2_ ← Y + Y*
4	*R_2_ ← R_1_ ∙ R_1_*	*Z_2_^2^ ← Y ∙ R_2_*
5	*⁕* *←**⁕* *+**⁕*	*Y_2_ ← Z_2_^2^ + Z_2_^2^*
6	*R_3_ ← X ∙ R_2_*	*R_3_ ← R_2_ ∙ Z*
7	*R_0_ ←Y_q_ ∙ Z^3^*	*R_2_ ←Y_2_ ∙ X*
8	*⁕* *←**⁕* *+**⁕*	*X_2_ ← X + R_0_*
9	*Z^3^ ←R_1_ ∙ R_2_*	*R_0_ ←R_1_ ∙ X_2_*
10	*R_2_ ← Z ∙ R_1_*	*R_1_ ← Z_2_^2^ ∙ Y_2_*
11	*X_3_ ←R_3_ + R_3_*	*X_2_ ← R_0_ + R_0_*
12	*X_3_ ← Z^3^ + X_3_*	*R_0_ ← R_0_ + X_2_*
13	*Z_3_^2^ ←(R_2_)^2^*	*X_2_ ← (R_0_)^2^*
14	*R_0_ ← R_0_ − Y*	*X_2_ ← X_2_ − R_2_*
15	*R_1_ ←(R_0_)^2^*	*Z_2_^2^ ← (R_3_)^2^*
16	*X_3_ ←R_1_ − X_3_*	*X_2_ ← X_2_ − R_2_*
17	*R_1_ ←R_3_ − X_3_*	*R_2_ ← R_2_ − X_2_*
18	*R_3_ ← R_1_ ∙ R_0_*	*Z_2_^3^ ← Z_2_^2^ ∙ R_3_*
19	*R_0_ ← Y ∙ Z^3^*	*Y_2_ ← R_0_ ∙ R_2_*
20	*Y_3_ ←R_3_ − R_0_*	*Y_2_ ← Y_2_ − R_1_*
21	*Z_3_ ← R_2_*	*Z_2_ ← R_3_*

Equation (A1): Four-segment Karatsuba multiplication formula (*m* is the length of a segment).

(A1) $$\begin{array}{l} A\times B=\left(A_{3}A_{2}A_{1}A_{0}\right)\times \left(B_{3}B_{2}B_{1}B_{0}\right)=\\ =\left(A_{3}\times 2^{3m}+A_{2}\times 2^{2m}+A_{1}\times 2^{1m}+A_{0}\right)\times \left(B_{3}\times 2^{3m}+B_{2}\times 2^{2m}+B_{1}\times 2^{1m}+\;B_{0}\right)=\\ =A_{0}B_{0}\times 2^{0}+\left(-A_{0}B_{0}-A_{1}B_{1}+\left(A_{0}+A_{1}\right)\left(B_{0}+B_{1}\right)\right)\times 2^{1m}+\\ \begin{array}{c} \begin{array}{l} +\left(-A_{0}B_{0}+A_{1}B_{1}-A_{2}B_{2}+\left(A_{0}+A_{2}\right)\left(B_{0}+B_{2}\right)\right)\times 2^{2m}+\\ +\left(\begin{array}{l} A_{0}B_{0}+A_{1}B_{1}+A_{2}B_{2}+A_{3}B_{3}-\left(A_{0}+A_{2}\right)\left(B_{0}+B_{2}\right)-\\ -\left(A_{0}+A_{1}\right)\left(B_{0}+B_{1}\right)-\left(A_{1}+A_{3}\right)\left(B_{1}+B_{3}\right)-\\ -\left(A_{2}+A_{3}\right)\left(B_{2}+B_{3}\right)+\\ +\left(A_{0}+A_{1}+A_{2}+A_{3}\right)\left(B_{0}+B_{1}+B_{2}+B_{3}\right) \end{array}\right)\times 2^{3m}+ \end{array} \end{array}\\ \begin{array}{c} \begin{array}{l} +\left(-A_{1}B_{1}+A_{2}B_{2}-A_{3}B_{3}+\left(A_{1}+A_{3}\right)\left(B_{1}+B_{3}\right)\right)\times 2^{4m}+\\ +\left(-A_{2}B_{2}-A_{3}B_{3}+\left(A_{2}+A_{3}\right)\left(B_{2}+B_{3}\right)\right)\times 2^{5m}+\\ +A_{3}B_{3}\times 2^{6m} \end{array} \end{array} \end{array}$$
